# Non-Targeted Lipidomics Analysis of Characteristic Milk Using High-Resolution Mass Spectrometry (UHPLC-HRMS)

**DOI:** 10.3390/foods14122068

**Published:** 2025-06-12

**Authors:** Tingting Wei, Tianxiao Zhou, Shenping Zhang, Zhexue Quan, Yang Liu

**Affiliations:** 1 Key Laboratory of Milk and Dairy Products Detection and Monitoring Technology, State Administration for Market Regulation, Shanghai Institute of Quality Inspection and Technical Research, Shanghai 200233, China; weitingting@fudan.edu.cn (T.W.); luciferztx@sjtu.edu.cn (T.Z.); zhangsp1@sqi.org.cn (S.Z.); 2 Fudan Microbiome Center, School of Life Sciences, Fudan University, Shanghai 200438, China; quanzx@fudan.edu.cn

**Keywords:** characteristic milk, lipidomics, UHPLC-HRMS, lipid composition, triacylglycerol, phospholipids, functional lipids

## Abstract

Milk lipids are fundamental to the nutritional quality, functional properties, and processing behavior of dairy products. In this study, we employed an untargeted lipidomics approach based on ultra-high-performance liquid chromatography coupled with ultra-high-performance liquid chromatography–high-resolution mass spectrometry (UHPLC-HRMS) to systematically characterize the lipid profiles of ten milk types from eight animal species, including camel, mare, donkey, goat, buffalo, yak, Jersey, and Holstein. A total of 640 lipid species were identified, spanning triglycerides (TGs), phospholipids (PLs), sphingolipids (SPs), ceramides (Cer), wax esters (WEs), and other subclasses. A statistical analysis revealed significant differences in lipid types and abundances among the milk samples. Camel milk exhibited the highest lipid diversity, with notable enrichment in phospholipids and sphingolipids, conferring superior emulsifying properties and stability. Mare milk was rich in polyunsaturated fatty acids (PUFAs), such as linoleic acid and alpha-linolenic acid, making it ideal for developing health-focused dairy products. Despite having the lowest total lipid content, donkey milk was enriched in cholesterol esters and PUFA, suitable for low-fat functional dairy products. Goat milk featured a balanced lipid composition with higher levels of medium-chain fatty acids (MCFAs), promoting digestibility. Buffalo milk was characterized by high TG and wax ester (WE) levels, offering high energy density and suitability for rich dairy products. Yak milk contained higher levels of ceramides (Cer) and saturated fatty acids, reflecting adaptations to high-altitude environments. Jersey milk and Holstein milk displayed similar lipid profiles, with stable compositions suitable for versatile dairy product development. Principal component analysis (PCA), hierarchical clustering, and volcano plot analyses further confirmed species-specific lipidomic signatures and revealed several potential lipid biomarkers, such as LPC (O-16:0) in Hongyuan yak milk, suggesting applications in geographical indication (GI) traceability. This study offers a comprehensive lipidomic landscape across diverse milk sources, providing molecular insights to guide the development of tailored, functional, and regionally branded dairy products.

## 1. Introduction

Dairy products account for approximately 14% of global agricultural trade, with milk being the largest commercial dairy product. Milk not only provides a stable source of nutrition for humans but also plays a crucial role in supporting growth and development [1]. Milk lipid, as one of the three primary nutritional components of dairy products, serves as an essential energy source and is closely associated with bone development, immune regulation, and overall health [2]. Lipids, as diverse biomolecules, possess rich structures and complex functions [3,4]. Chemically, lipids are categorized into eight major classes: fatty acids (FAs), glycerolipids (GLs), glycerophospholipids (GPs), sphingolipids (SPs), sterol lipids (ST), saccharolipids (SLs), prenol lipids (PR), and polyketides (PKs). The Lipid MAPS Structure Database (LMSD, https://www.lipidmaps.org) contains over 40,000 lipid entries based on these classes [5].

Functional lipids in milk, such as medium-chain triglycerides (MCTs), polyunsaturated fatty acids (PUFAs), phospholipids, and sphingolipids, significantly contribute to infant development, immunity enhancement, and metabolic regulation [6]. Additionally, milk fat globule membranes (MFGMs), composed of triglycerides (TGs), phospholipids, sphingolipids, and membrane-bound proteins (mainly glycoproteins), show potential benefits in gut health, cardiovascular health, osteoporosis prevention, and cognitive function [7,8]. However, while lipids have clear health benefits, some, such as saturated fatty acids (SFAs) at high concentrations, may increase cardiovascular disease risk, whereas PUFA can help mitigate this risk and boost immunity [9]. The composition and characteristics of milk lipid significantly affect the flavor, texture, and stability of dairy products, directly influencing consumer sensory experiences and product quality. Hence, comprehensive lipid profiling is crucial for developing high-quality liquid milk products and meeting modern consumer demands [4].

Milk typically contains 3–5% lipid, of which 98% consists of TGs, while polar lipids such as GPs and SPs account for only 0.5–1%. Although approximately 400 milk lipids have been identified, many low-abundance lipids remain undiscovered [10]. As key components of biological processes, milk lipids exhibit complex and diverse structures, posing significant analytical challenges [11]. Particularly for identifying and quantifying low-concentration compounds and distinguishing lipid isomers, advanced techniques such as high-resolution mass spectrometry (HRMS) have proven to be highly advantageous and have become the mainstream method in non-targeted lipid research [12].

With the introduction of “omics” and an increasing understanding of lipids’ critical biological functions, lipidomics has emerged as a cutting-edge research field offering new perspectives on milk lipid functionality and quality. However, there is no standardized method to comprehensively characterize all lipids. High-performance liquid chromatography–mass spectrometry (HPLC-MS) is widely used for analyzing TG molecules and isomers in milk lipid [13]. Matrix-assisted laser desorption ionization–time of flight mass spectrometry (MALDI-TOF MS) is employed for the molecular characterization of milk lipids [14], while gas chromatography–mass spectrometry (GC-MS) is extensively applied to separate and identify milk fatty acids [15]. MS data can be cross-referenced with the LipidSearch database to identify specific lipids [16], providing a convenient and reliable approach for milk lipidomics. Despite their broad applications, these methods face challenges due to the complexity of lipid species, limitations in MS instrumentation, and the influence of origin, climate, and feeding conditions on milk composition.

While lipidomics has been widely applied in studying cow milk and common dairy products, systematic research on characteristic milks (e.g., camel, mare, donkey, and goat milk) remains limited. These milks may exhibit significant differences in lipid types, distributions, and functional properties, but targeted analyses are lacking. Non-targeted lipidomics combined with high-resolution mass spectrometry enables the comprehensive profiling of milk lipids, uncovering their types, distributions, and functional characteristics while identifying potential differential lipid biomarkers to support customized dairy product development. In this study, ultra-high-performance liquid chromatography–high-resolution mass spectrometry (UHPLC-HRMS) was used to perform non-targeted lipidomics on camel milk, mare milk, donkey milk, goat milk, buffalo milk, yak milk, Jersey milk, and Holstein milk. The analysis revealed differences in lipid composition and distribution across these milks. The selection of these milk types was based on species diversity and their increasing relevance to human consumption. For example, mare and donkey milk are low-fat and rich in PUFA, making them ideal for health-conscious or special-diet populations. Camel milk, particularly popular in regions like Xinjiang, is considered a potential substitute for human milk due to its bioactive components. Goat milk is valued for its low allergenicity and digestibility. Buffalo milk and Jersey milk are known for their high lipid content and rich flavor, catering to consumers who prefer creamy and full-bodied dairy products. This research aims to provide critical data for elucidating the functional properties, nutritional value, and quality control of characteristic milks, supporting the development of high-quality, targeted dairy products.

## 2. Materials and Methods

### 2.1. Materials

#### 2.1.1. Sample Collection

This study collected raw milk samples from ten different species, with six replicates for each species, including camel, mare, S4—donkey, S5—goat, S6—buffalo, S7—Jersey, S8—Holstein and yak milk. The camel milk (S1—camel1 and S2—camel2) samples were obtained from two different dairy factories in Xinjiang, while mare (S3) and goat (S5) milk were sourced from Xinjiang. Donkey (S4) and Jersey (S7) milk were collected from different dairy factories in Hebei. Buffalo (S6) milk was sourced from Guangxi, regular yak milk (S9-Yak1) from Qinghai, Hongyuan yak milk (S10-Yak2) from Sichuan, and Holstein (S8) milk was obtained from Inner Mongolia. All samples were collected under sterile conditions, transported to the laboratory in a refrigerated state, and stored at −80 °C until analysis.

The inclusion of two sources of camel (S1, S2) and yak milk (S9, S10) was intentional, aiming to assess potential intraspecies lipidomic variation. Notably, S10 corresponds to Hongyuan yak milk, a nationally recognized geographical indication (GI) product in China. This design enabled us to investigate whether specific lipid species could serve as molecular markers for regional origin authentication or the detection of adulteration in GI-protected dairy products.

#### 2.1.2. Reagents and Consumables

Chromatography-grade methanol, acetonitrile, isopropanol, dichloromethane, and chloroform (Thermo Fisher Scientific, Waltham, MA, USA); analytical-grade ammonium formate (Sigma-Aldrich, St. Louis, MO, USA); ultrapure water: produced using a Milli-Q system (Millipore, Billerica, MA, USA); Lipidmix (SPLASH™ LIPIDOMIX™, Avanti Polar Lipids, Alabaster, AL, USA), distributed by Merck KGaA (Darmstadt, Germany); Accucore™ C18 column (2.6 μm, 2.1 × 150 mm, Thermo Fisher Scientific, USA).

#### 2.1.3. Instruments

UHPLC-HRMS system (Thermo Fisher Scientific, USA); MS104S electronic balance (precision: 0.0001 g, Mettler Toledo, Greifensee, Switzerland); Reax Control vortex mixer (Heidolph, Schwabach, Germany); Centrifuge 5804 (Eppendorf, Hamburg, Germany); nitrogen evaporator (Anpel Laboratory Technologies, Shanghai, China).

### 2.2. Methods

#### 2.2.1. Total Lipid Extraction

The total lipid extraction method was based on the Bligh and Dyer method [17,18], with modifications as follows: 2 mL of raw milk was added to an Eppendorf^®^ 15 mL centrifuge tube (made of solvent-resistant polypropylene), followed by 4 mL of methanol and 1.6 mL of water. The mixture was vortexed thoroughly. Then, 4 mL of chloroform was added, and the mixture was incubated at room temperature for 30 min to ensure lipid release into the organic phase. The mixture was centrifuged at 2000 r/min for 15 min, and the lower organic layer was separated. Subsequently, 4 mL of chloroform was added to the remaining aqueous phase, and the extraction was repeated twice. All three organic phases were combined and transferred to a pre-weighed, oven-dried centrifuge tube. The solvent was evaporated using a nitrogen evaporator at room temperature, and the sample was further dried in an 80 °C oven to a constant weight. The final tube weight was recorded, and the difference before and after drying represented the total lipid content in 2 mL of raw milk.

#### 2.2.2. Sample Preparation

Each milk sample was thawed and mixed thoroughly before use. An 80 μL aliquot was placed in an Eppendorf^®^ 1.5 mL EP tube (made of solvent-resistant polypropylene), followed by the addition of 10 μL of internal standard solution (lipidmix) for normalization. Then, 200 μL of cold methanol and 80 μL of distilled water were added, and the mixture was vortexed for 1 min. Next, 200 μL of chloroform was added, and the sample was incubated at room temperature for 30 min. The mixture was centrifuged at 2000 r/min for 15 min, and the lower organic layer was collected. The extraction was repeated twice on the remaining aqueous phase, and the organic phases were combined. The pooled organic phase was evaporated to dryness using a nitrogen evaporator and then reconstituted in 200 μL of dichloromethane/methanol (1:1, *v*/*v*) for UHPLC-HRMS analysis.

#### 2.2.3. UHPLC-HRMS Analysis

Lipidomic profiling was performed using a Thermo Scientific™ Q Exactive™ high-resolution mass spectrometer coupled with a UHPLC system. Chromatographic separation was conducted using an Accucore™ C18 column (150 × 2.1 mm, 2.6 μm) under a gradient elution program. The mobile phases consisted of Phase A (60% acetonitrile, 40% water with 10 mM ammonium formate) and Phase B (90% isopropanol, 10% acetonitrile with 10 mM ammonium formate). The flow rate was set at 250 μL/min, and the column temperature was maintained at 45 °C.

The gradient elution program was as follows:

0–1 min: A 70%, B 30%;

1–3 min: A decreased to 20%, B increased to 80%;

3–26 min: A reduced to 10%, B increased to 90%;

26–36 min: A 0%, B 100%;

36–38 min: isocratic elution;

38–40 min: A 70%, B 30%.

The injection volume was 2 μL. Mass spectrometry was conducted using an electrospray ionization (ESI) source in both positive- and negative-ion modes. The ion source temperature was set to 300 °C and the ion transfer tube to 350 °C. Spray voltages were set at 3.0 kV (positive) and 2.5 kV (negative). The sheath, auxiliary, and sweep gas flow rates were set at 45, 15, and 1 arb, respectively. The MS1 scan range was 200–1800 *m*/*z* with a resolution of 70,000 at *m*/*z* 200, and the MS2 resolution was 17,500. For each full scan, 10 fragment spectra were acquired to ensure comprehensive lipid identification.

#### 2.2.4. Data Processing

LipidSearch 5.0 (Thermo Scientific™) was used for lipid identification and processing, including raw data parsing, peak alignment, retention time correction, and peak area extraction. The following settings were applied: MS1 tolerance, 5 ppm; MS2 tolerance, 10 ppm; filter settings, (Rank = 1) and (Grade = “A” or Grade = “B” or (Grade = “C” and IDScore >= 0.5); RT Tol (min), 0.15; calc method, median. Subsequently, the trial version of SIMCA 17.0, MetaboAnalyst 5.0, and R were employed for multivariate statistical analyses. Missing values caused by low-abundance lipids were imputed using the limit of detection (LOD × 0.5) method. Statistical analyses included principal component analysis (PCA) and differential lipid screening (criteria: VIP > 1, *p* < 0.01). Data visualization was performed using heatmaps and volcano plots to comprehensively explore lipid variation among samples.

## 3. Results

### 3.1. Reliability of Methods and Results

A pooled milk sample was prepared by mixing equal volumes of the six replicate samples from ten species to serve as a quality control (QC) sample. The specific results are shown in Table S1. This QC sample was used to monitor the stability of the instrumentation and ensure data reliability. The ion chromatograms of the QC samples exhibited high reproducibility, indicating excellent data stability. Moreover, the QC samples demonstrated high correlation between positive- and negative-ion modes, further confirming the quality and reliability of the data. These findings validate the analytical methods employed in this study.

### 3.2. Differences in Total Lipid Content of Raw Milk Samples

**Figure 1:**
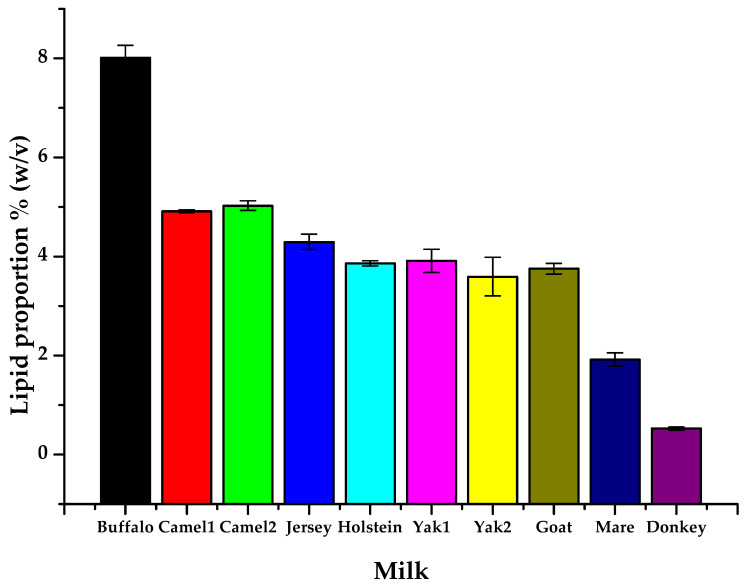
Differences in lipid content among different raw milk types.

An analysis of the lipid content across various raw milk samples (Figure 1) revealed significant differences, which have critical implications for the nutritional value and applications of these dairy products. Specifically, buffalo milk exhibited the highest lipid content at 8.01%, while camel milk also had relatively high lipid levels, at 4.91% and 5.03%. Jersey and Holstein milk showed moderate lipid levels, with 4.30% and 3.86%, respectively. Goat milk and yak milk displayed comparatively lower lipid content, at 3.75% and 3.59–3.91%, respectively. Mare milk and donkey milk had extremely low lipid content, with donkey milk nearly devoid of lipids.

### 3.3. Differences in Total Lipid Composition of Raw Milk Samples

This study utilized UHPLC-HRMS combined with the LipidSearch 5.0 database to profile 640 lipid species, uncovering significant compositional differences between various characteristic milks and conventional Holstein milk. These differences have a direct impact on the nutritional value, sensory characteristics, and processing suitability of dairy products.

**Figure 2:**
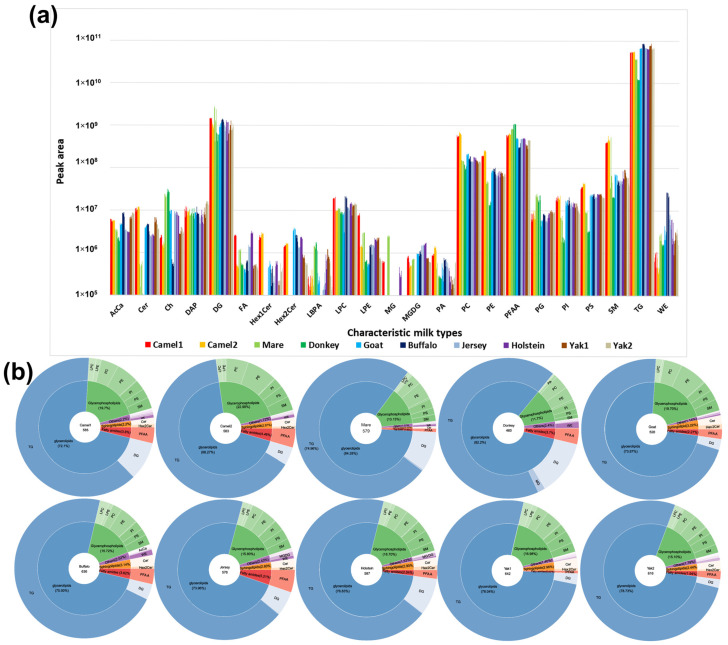
Lipid composition differences among various raw milk types identified by Lipidsearch. Differences in lipid composition among different raw milk types based on peak area (**a**). The distribution of lipid subclasses and categories (**b**). In (**b**), the central circle represents the species of raw milk origin and the total number of identified lipids, the middle ring indicates the proportion of major lipid categories, and the outer ring illustrates the distribution of key lipid subclasses. The abbreviations used in this study are as follows: ACCa—Acyl carnitine; Cer—ceramide; Ch—cholesterol; DAP—Dialkyl phthalate; DG—Diglyceride; FA—fatty acid; Hex1Cer—Hexosyl ceramide; Hex2Cer—Dihexosyl ceramide; LBPA—Monoacylglycerophosphomonoradylglycerols; LPC—Lyso phosphatidylcholine; LPE—Lyso phosphatidylethanolamine; MG—Monoglyceride; MGDG—Monogalactosyl diacylglycerol; PA—Phosphatidic acid; PC—Phosphatidylcholine; PE—Phosphatidylethanolamine; PFAA—Primary amides; PG—Phosphatidylglycerol; PI—Phosphatidylinositol; PS—Phosphatidylserine; SM—sphingomyelin; TG—Triacylglycerol; WE—wax ester (hereafter referred to as such).

Figure 2a presents the total peak area of identified lipid species across various raw milk types, reflecting their relative abundance. The x-axis denotes different lipid classes, while the y-axis (logarithmic scale) represents the corresponding peak area, facilitating a comparative assessment of lipid composition among milk types. Camel milk exhibited higher concentrations of TG, PC, PE, PI, PS, SM, and Cer. Mare milk showed notable levels of DG, cholesterol, LBPA, MG, PG, and SM. Donkey milk demonstrated unique lipid characteristics, with low total lipid content but relatively high levels of cholesterol esters, LBPA, and PFAA, while key lipids like WE, PI, PA, MG, and Cer were present in much lower concentrations, with Cer and MG being nearly undetectable in some samples. Goat milk displayed a balanced lipid composition, with consistently high levels of most lipids except MG. The contents of TG and WE were more abundant in buffalo milk, with negligible Ch. Jersey milk and Holstein milk exhibited similar lipid profiles, with nearly identical lipid content except for LBPA. Yak milk showed similarities to donkey and goat milk, characterized by low FA levels and undetectable MG, but exhibited exceptional balance in other lipids, with notably high SM content second only to camel milk.

Figure 2b provides a detailed breakdown of lipid composition across different raw milk types. The central circle denotes the milk source species and the total number of identified lipid species. The middle ring presents the proportion of major lipid categories, including GLs, GPs, sphingolipids, fatty amides, and other minor lipid classes. The outer ring further refines these categories into lipid subclasses, illustrating the specific distribution patterns of key lipids such as PC, PE, SM, and others. This visualization highlights both the diversity and predominance of lipid species in different milk types, offering insights into their potential nutritional and functional implications. Consistent with previous studies, GLs, particularly TGs, were the most abundant lipid class in milk (including human milk) [19], followed by GPs [20]. Compared to Holstein milk, characteristic milk types exhibited a greater diversity of LPE species. Notably, mare and donkey milk contained fewer LPC and LPE species but had a higher proportion of TGs, suggesting that their reduced lipid content may be concentrated in non-TG lipid classes.

### 3.4. Multidimensional Statistical Analysis of Different Characteristic Milks

**Figure 3:**
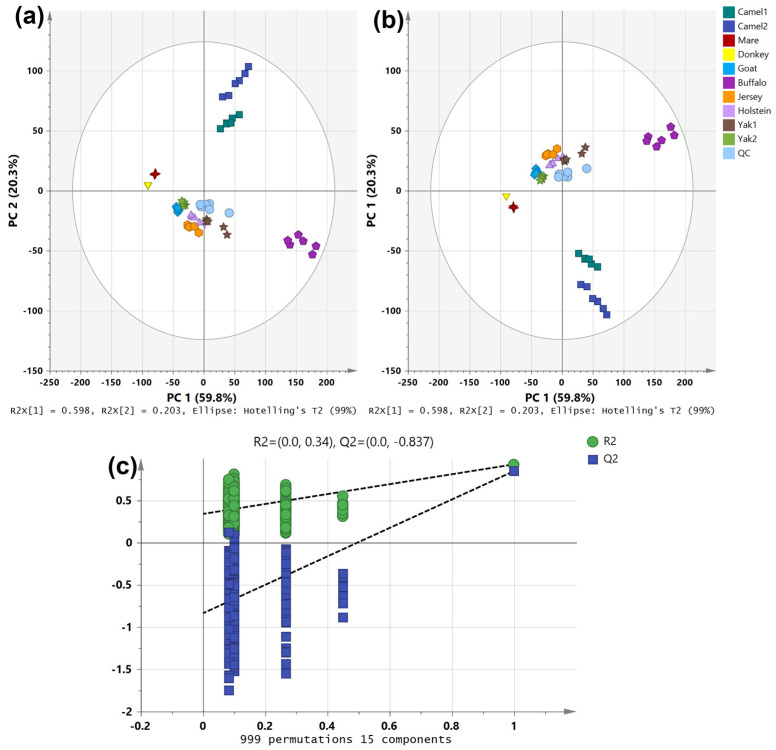
Two-dimensional PCA score map (**a**), corresponding validation by PLS-DA (**b**), and permutation tests with 999 iterations (**c**) of different characteristic milk samples.

PCA and partial least squares—discriminant analysis (PLS-DA) were employed to investigate the lipidomic variations among different raw milk samples. As shown in Figure 3a, PCA score plots revealed distinct clustering patterns, indicating significant differences in lipid composition. Camel milk (camel1 and camel2) exhibited a high degree of similarity, forming a well-defined cluster that was clearly separated from other milk samples, reflecting its unique lipid profile. Donkey and mare milk samples clustered closely together, suggesting similar lipid compositions. In contrast, buffalo milk was distinctly positioned, indicating significant deviations in lipid composition compared to other milk types.

To further validate these distinctions, PLS-DA analysis was conducted (Figure 3b), which demonstrated a clear separation among different milk samples, confirming the robustness of the classification based on lipid composition. Yak milk (Yak1 and Yak2) and camel milk displayed particularly distinct lipidomic profiles compared to other milk types, further emphasizing their unique biochemical characteristics. The QC samples clustered near the origin, indicating the high reproducibility and reliability of the analytical workflow.

Additionally, a permutation test with 999 iterations was performed (Figure 3c) to assess the model’s validity. The results showed an R^2^ value of 0.34 and a Q^2^ value of −0.837, indicating that the PLS-DA model was not overfitted and that the observed classification was statistically reliable [21]. The variance captured by PC1 (59.8%) and PC2 (20.3%) suggests that these two principal components effectively account for the major lipidomic differences among the raw milk samples.

**Figure 4:**
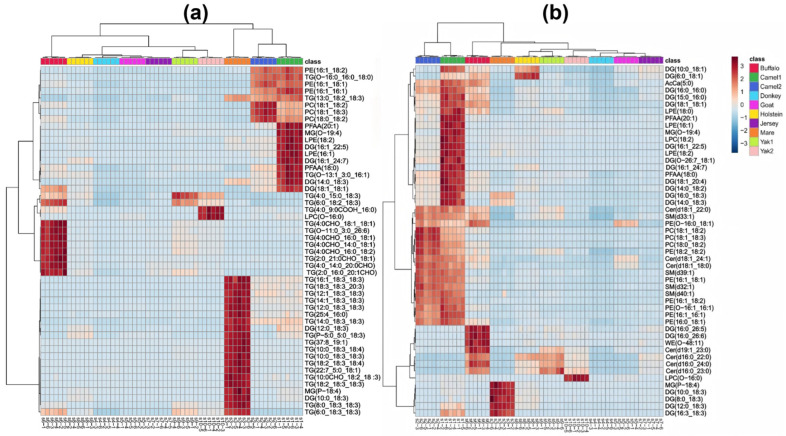
Heatmap analysis of lipid composition differences among different raw milk types. Hierarchical clustering heatmaps illustrating the top 50 significantly different lipids among the 640 identified lipids (**a**) and 135 non-TG lipids (**b**) across various raw milk types.

The hierarchical clustering analysis reveals distinct patterns in lipid composition among different milk types. Samples were grouped based on lipid characteristics, reflecting metabolic similarities and variations. The differences in the number and type of lipid molecules contributed to the clustering distance between samples, indicating that certain milk types shared more similar lipid profiles than others. Camel milk (camel1 and camel2) exhibited a unique lipid profile, characterized by a higher abundance of MG, DG, SM, PE, PC, LPC, LPE, and PFAA, compared to other milk types. Camel milk was particularly rich in phospholipids (PC, PE, LPC, and LPE), while its TG content and diversity were relatively lower among the top 50 significantly different lipids (Figure 4a). The clustering patterns observed in Figure 4a strongly corroborate the grouping tendencies revealed by the PCA results in Figure 3a, indicating consistency between unsupervised clustering and variance-based dimensionality reduction. Specifically, the two camel milk samples (camel1 and camel2) were tightly clustered in both analyses and clearly separated from other groups, primarily due to their elevated levels of SM and PC. Buffalo milk exhibited the greatest distance from other bovine milks (e.g., Holstein, Jersey, and yak) in both PCA space and hierarchical clustering, reflecting its distinct lipidomic profile characterized by high TG and WE content. Although mare milk was rich in PUFA, it did not cluster closely with donkey milk in Figure 4a—likely because of the markedly lower total lipid content in donkey milk (Figure 1), which weakened their overall lipidomic similarity despite partial overlap in the PCA score plots.

In contrast, Holstein, donkey, and mare milk displayed significantly lower sphingolipid content. The lipid composition of camel milk was also characterized by long-chain polyunsaturated fatty acids (PUFAs) [22], while containing lower levels of short-chain fatty acids [23], suggesting a distinct metabolic adaptation. Buffalo milk exhibited a high concentration of TG, composed of short-, medium-, and long-chain fatty acids, providing a rich energy source. It also contained long-chain PUFAs (e.g., C18:1, C18:2, C18:3, C20:1A, and C26:6) but was relatively lower in phospholipids and lyso phospholipids compared to camel milk. The clustering results indicated that buffalo milk was distinct from bovine milks (Holstein and yak milk) and also significantly different from camel milk, suggesting that buffalo milk has the most distinct lipid composition among all milk types analyzed. Additionally, buffalo milk contained higher Cer levels than other milk types, along with DG species (e.g., DG (16:0_26:5) and DG (16:0_26:6)) that had higher degrees of unsaturation (Figure 4b). Combined with Figure 2b, buffalo milk exhibited the highest diversity of Cer species, and Figure 2a also showed that its WE content and diversity were greater than other characteristic milk types. These findings suggest that Cer and WE could serve as potential lipid biomarkers, distinguishing buffalo milk from other milk types.

Mare milk had a relatively low total lipid content (Figure 1) but was rich in medium- and long-chain PUFAs, particularly C18:2 and C18:3 (linolenic and linoleic acid-essential for human health). Its TG and DG molecules were primarily composed of PUFA such as C18:3, C18:4, and C20:3 (Figure 4). While PCA (Figure 3a) showed that donkey and mare milk shared similar lipid compositions, mare milk had significantly higher lipid content than donkey milk. Moreover, donkey milk showed no significant enrichment among the top 50 differential lipids, aligning with its simpler lipidomic profile (Figure 2). A comparative analysis of lipid composition between Hongyuan yak milk and regular yak milk revealed similar metabolic characteristics. However, Hongyuan yak milk was richer in LPC (O-16:0) and TG (6:0_9:0COOH_16:0), including saturated fatty acids of varying chain lengths, whereas regular yak milk contained more unsaturated TG (e.g., C18:2 and C18:3). Goat milk exhibited low total lipid content without notable class-specific enrichment, contrasting sharply with the complex TG/phospholipid/PUFA profiles of camel, buffalo, and mare milk. Its lipidome was less distinct compared to Jersey, Holstein, or donkey milk.

**Figure 5:**
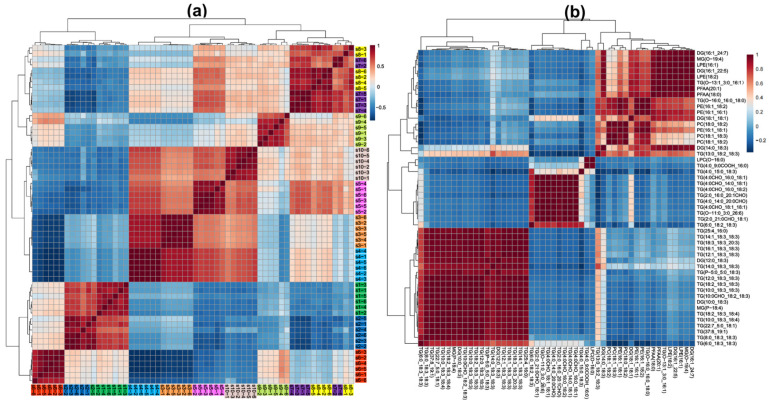
A correlation analysis of raw milk samples (**a**) and the top 50 differential lipid molecules across characteristic milk samples (**b**).

The correlation analysis chart provides insights into the relationships among different raw milk samples and the metabolic connections between key lipid molecules. In Figure 5a, the clustering patterns among samples were distinct and closely aligned with the PCA results (Figure 3a). The two camel milk samples formed a separate cluster, which further grouped with buffalo milk, creating a unique cluster that was clearly differentiated from other characteristic milk types. This indicates that camel and buffalo milk share certain lipid metabolic characteristics that set them apart from other milk types. Additionally, Holstein and Jersey milk exhibited similar clustering patterns with relatively low inter-group differentiation, suggesting that their lipid compositions might be closely related (Figure 2a and Figure 5a).

Figure 5b further explores the metabolic relationships between lipid molecules, revealing significant positive and negative correlations among the top 50 differential lipids. These correlations reflect the functional differentiation and synergistic roles of lipid molecules in milk lipid metabolism. Notably, a strong negative correlation was observed between lyso phosphatidylcholine—LPC (O-16:0)—and several TGs, suggesting potential functional differentiation in metabolic pathways. Additionally, strong positive correlations among multiple TG molecules indicate their cooperative role in lipid storage and energy regulation. This indicates that LPC and TG may have functional differentiation in metabolic pathways, with LPC being more involved in maintaining cell membrane stability and signal transduction, while TG primarily serves as an energy reservoir. When energy demand increases in milk lipid metabolism, TG synthesis becomes more active, and LPC metabolism decreases relatively, and vice versa. This highlights a complementary metabolic relationship between phospholipids and TG. Secondly, many TG molecules exhibit strong positive correlations with each other, indicating that these TG may work synergistically to support energy supply and lipid storage, particularly in regulating energy density and improving the texture of dairy products. The cooperative action between highly saturated and polyunsaturated TG helps maintain the metabolic balance of milk lipids. Additionally, MG and DG show significant positive correlations with phospholipids (e.g., PC and PE) but negative correlations with TG. MG and DG act as intermediate metabolites in TG metabolism and are also precursors for phospholipid synthesis. This suggests that when metabolism favors energy storage, MG and DG are rapidly converted into TG. Conversely, when structural and functional demands dominate, MG and DG facilitate the synthesis of phospholipids, enhancing their positive correlation with phospholipids. Finally, the correlation analysis reveals a negative correlation between phospholipids (e.g., PC and PE) and certain TGs, further supporting the functional independence of storage lipids and structural lipids in metabolic roles. Phospholipids are crucial for cell membrane stability and signal transduction, whereas TGs primarily support energy storage. This indicates that an increase in energy storage demand in milk lipid metabolism may lead to reduced phospholipid metabolic activity, emphasizing the complementary roles of these two lipid types in metabolic regulation. Alternatively, phospholipids might predominantly participate in maintaining cell membrane structures rather than energy storage. This dynamic balance reflects the regulatory capacity of milk lipid metabolism to meet both energy demands and structural–functional requirements.

**Figure 6:**
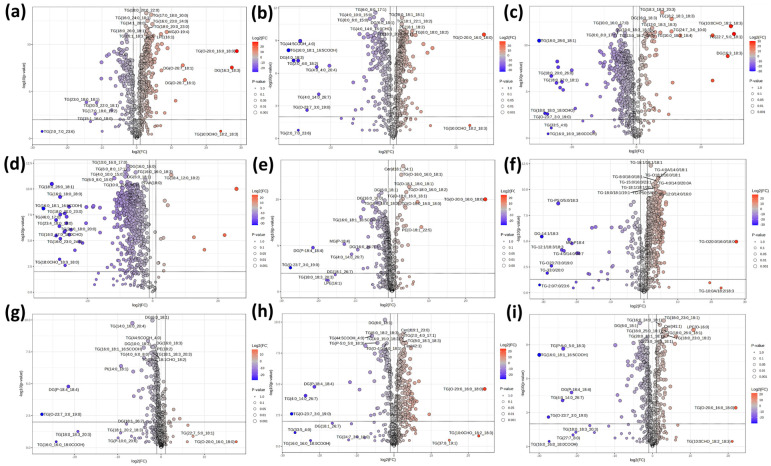
Volcano plots of lipidomic differences between various characteristic milk samples and Holstein milk: camel1 (**a**); camel2 (**b**); mare (**c**); donkey (**d**); goat (**e**); buffalo (**f**); Jersey (**g**); yak1 (**h**); yak2 (**i**). Each plot illustrates the fold change (log_2_FC) and statistical significance (–log_10_*p*-value) of 640 identified lipid species between the indicated characteristic milk and Holstein milk, with thresholds set at *p* < 0.01 and |fold change| > 2. The lipids on the right side of each plot are significantly upregulated, while those on the left are significantly downregulated. The size and color of each point represent the statistical significance, with larger, darker points denoting more significant differences. The top 20 most significantly different lipid species are labeled in each plot for clarity. The same criteria and labeling strategy are applied throughout.

Our comparative analysis revealed substantial lipidomic divergence between characteristic milks and Holstein controls. Camel milk exhibited the most pronounced enrichment in structural lipids. Specifically, camel1 and camel2 showed 444 (293 upregulated) and 456 (274 upregulated) differential lipid species, respectively, with markedly elevated levels of GP—such as LPC (O-16:0) and PE (O-16:0_18:1)—as well as sphingomyelins (e.g., SM (d40:1)) and Cer (e.g., Cer (t42:1)) (Figure 6a,b). This profile suggests enhanced membrane plasticity and signaling capacity, potentially reflecting evolutionary adaptations to arid environments. The concurrent downregulation of specific TG further indicates a shift from energy storage to functionally active lipid classes, which may contribute to camel milk’s bioactivity and thermal resilience.

Mare milk also displayed striking divergence, with 479 differentially expressed lipids. It was characterized by the selective upregulation of PUFA-rich TG and DG species, notably linoleic acid (C18:2) and α-linolenic acid (C18:3), which are essential for human health. This is consistent with Figure 2, where PUFA-enriched lipid classes were prominent, potentially contributing to mare milk’s nutritional benefits, oxidative susceptibility, rapid acidification, and distinctive flavor profile (Figure 6c). In contrast, donkey milk presented an extreme case of lipid depletion, with 548 out of 640 lipid species significantly downregulated and only a minimal upregulation of PUFA-containing TG (Figure 6d). This reflects both its low total lipid content and narrow lipid diversity (Figure 1 and Figure 2).

Among the bovine-derived milks, Jersey milk demonstrated the least variation from Holstein, with only 106 differential lipid species identified (6 upregulated, 100 downregulated; Figure 6g). The few upregulated lipids were limited to a subset of TG and DG, whereas the majority of downregulated species were glycerides, including TG, DG, and PI. This minimal divergence reflects the close phylogenetic and breeding relationship between Jersey and Holstein cows and indicates a high degree of metabolic conservation, thereby limiting the potential for identifying distinctive lipid biomarkers. In contrast, buffalo milk—though also bovine—displayed a markedly divergent lipid profile, with 402 lipid species significantly altered, including 339 upregulated (Figure 6f). Notably, buffalo milk was enriched in long-chain saturated TG and WE, consistent with a more energy-dense lipid composition compared to Holstein milk. As further illustrated in Figure 2, the WE content in buffalo milk was substantially higher, suggesting its value as a species-specific marker for energy-related lipid functionality and compositional authentication in dairy applications. Further divergence was observed in yak milk, which exhibited distinctive lipidomic traits likely linked to high-altitude adaptation. These included elevated levels of odd-carbon TGs (e.g., TG (26:0_18:1_19:1)) and ceramides (Figure 6h,i), potentially reflecting unique dietary inputs or physiological responses to hypoxic environments. Although yak milk is also of bovine origin, its lipid composition was clearly distinct from that of both Holstein and buffalo milks, underscoring the influence of regional and environmental factors on milk lipid profiles.

Lastly, goat milk presented a metabolically contrasting profile, showing a clear preference for ceramide synthesis over GP accumulation (Figure 6e). This lipid routing pattern may relate to its enhanced digestibility and biofunctional properties in human nutrition. In particular, goat milk showed a metabolic advantage in Cer and SFA synthesis pathways, which could serve as potential lipid-based biomarkers distinguishing it from Holstein milk.

**Figure 7:**
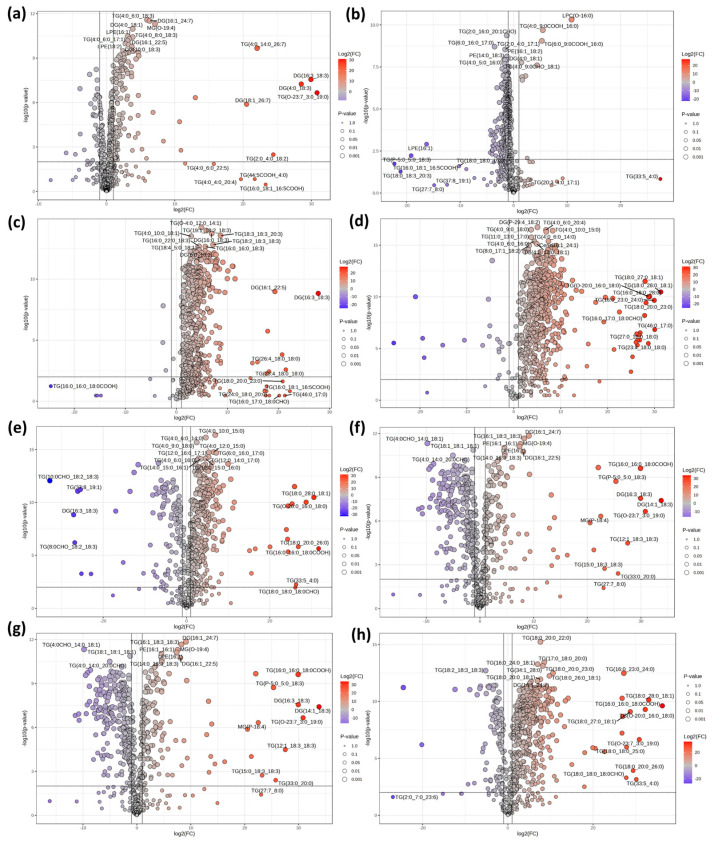
Volcano plots of pairwise lipidomic comparisons among different raw milk types. Camel1 vs. camel2 (**a**); yak1 vs. yak2 (**b**); mare vs. donkey (**c**); goat vs. donkey (**d**); goat vs. mare (**e**); camel1 vs. buffalo (**f**); camel1 vs. goat (**g**); camel1 vs. mare (**h**).

Figure 7a shows minimal lipidomic differences between camel1 and camel2, with 164 significantly altered lipids—131 upregulated and 33 downregulated—likely due to their shared species origin. The significantly upregulated lipids are mainly GLs, covering many short-chain saturated fatty acids. The significantly downregulated lipids are also primarily GLs, with a focus on medium- to long-chain saturated fatty acids. Both camel milk samples originate from Xinjiang, and their differences are limited to certain GLs. As shown in the PCA plot in Figure 3, the two samples are closely positioned and far from other species’ milk samples. This suggests that these lipid differences may be due to variations in feeding environments and are unlikely to serve as potential differential markers. Among the 640 lipid components, 262 showed significant differences between Hongyuan yak milk and regular yak milk, with only 9 upregulated and 253 downregulated lipids (Figure 7b). The upregulated lipids include LPC and TG. Among these lipids, LPC (O-16:0) stands out as a lipid component significantly differentiating Hongyuan yak milk from regular yak milk, making it a potential differential marker.

Figure 7c presents the volcano plot comparing mare milk and donkey milk. Despite their close proximity on the PCA plot, 463 out of 640 lipid components show significant differences, with 460 significantly upregulated and only 3 significantly downregulated. The upregulated lipids include TG, DG, and Cer. Notably, the elevated Cer levels serve as a key marker distinguishing mare milk from donkey milk, positioning it as a potential differential marker. The downregulated lipids consist of only four components: TG (18:0_18:1_18:1COOH), DG (P-50:6), PE (O-18:1_22:5), and TG (6:0_18:0_17:1). These further highlight that donkey milk has significantly lower lipid content and diversity compared to other types of milk.

Figure 7d shows the volcano plot comparing goat milk and donkey milk. Out of 640 lipid components, 551 show significant differences, with 530 upregulated and 21 downregulated. The upregulated lipids are primarily TG and Cer, mostly composed of long-chain saturated fatty acids. The downregulated lipids, including TG and DG, are primarily unsaturated fatty acids. These lipids may serve as potential differential markers between goat milk and donkey milk.

Among the 640 lipid components compared between goat milk and mare milk, 450 show significant differences, with 339 upregulated and 111 downregulated (Figure 7e). The upregulated lipids are primarily saturated fatty acid glycerides, while the downregulated lipids are mainly enriched in unsaturated fatty acids. Together with Figure 6c, these results further indicate that mare milk is rich in PUFA, particularly linoleic acid and linolenic acid, which may serve as potential differential markers.

As the PCA plot indicates that buffalo milk is more distantly clustered from other milk types, Figure 7f presents the volcano plot comparing camel1 and buffalo milk. Of the 640 lipid components, 381 show significant differences, with 141 significantly upregulated and 240 downregulated. The upregulated lipids are mainly long-chain polyunsaturated fatty acid glycerides, along with certain phospholipids and ceramides. In contrast, the downregulated lipids are predominantly short-chain fatty acid glycerides, such as TG (2:0_4:0_18:2), TG (4:0_6:0_12:0), and TG (4:0_4:0_18:3). These results suggest that camel milk has a greater capacity for storing long-chain fatty acids, whereas buffalo milk is enriched in short-chain fatty acids. Among the 640 lipid components compared between camel and goat milk, 445 showed significant differences, with 352 upregulated and 93 downregulated (Figure 7g). The upregulated lipids include various GL, along with several phospholipids such as LPE (18:0), LPC (14:0), PE (16:1_16:1), SM (d38:1), PE (16:1_18:2), and SM (d40:1). In conjunction with the heatmap (Figure 4), these findings highlight camel milk’s enrichment in phospholipids, suggesting these species as potential differential markers. Among the 640 lipid components compared between camel1 and mare milk, 493 show significant differences, with 400 significantly upregulated and 93 downregulated (Figure 7h). Consistent with the previously described lipid profile of camel milk (Figure 4, Figure 6a,b, and Figure 7a,f,g), the upregulated lipids include saturated TG and a variety of phospholipids, further underscoring camel milk’s enrichment in phospholipids and supporting their role as potential differential markers. The downregulated lipids are mainly polyunsaturated TG. As noted earlier (Figure 4 and Figure 6c), mare milk is distinguished by its high levels of PUFA, particularly linolenic and linoleic acids.

## 4. Discussion

### 4.1. Comparative Lipid Content Across Species

Cow milk dominates global dairy production (83.1%), followed by buffalo (12.8%), goat (2.4%), sheep (1.4%), and camel milk (0.3%) [24]. However, increasing attention is being paid to non-bovine and region-specific milk types due to their unique nutritional, functional, and sensory characteristics. Consistent with previous reports [24,25,26], our results confirmed substantial interspecies variation in total lipid content. Among ruminants, buffalo milk displayed the highest lipid content (8.01%), exceeding earlier reports of up to 7.1%, while camel milk from two Xinjiang sources showed moderate lipid levels (4.91% and 5.03%), implying possible regional or genetic influences. In contrast, non-ruminant milks, especially donkey milk (0.52%) and mare milk, exhibited significantly lower lipid content, aligning with earlier findings that report donkey milk fat content typically ranges from 0.16% to 1.3% [27,28]. These data underscore the importance of species and origin selection in milk-based nutritional evaluation and functional food development.

### 4.2. Ruminant Milk: Lipid Diversity, Regionality, and Conservation

In this study, we identified over 640 lipid species using UHPLC-HRMS coupled with LipidSearch 5.0, exceeding the lipid coverage of many previous milk lipidomic investigations (Table 1). TGs were the dominant class in all milk types, but species-level differences in individual lipid molecules were striking (Figure 2). Unlike earlier research that focused on common milk types (e.g., cow, goat, camel), our study incorporated broader taxonomic and regional diversity, including geographically distinct samples such as camel1/2 and yak1/2. Notably, we observed that among the top 50 differential lipids, the differences between camel1 and camel2, and between yak1 and yak2 were relatively minor, whereas differences between species were markedly greater. This suggests a high degree of lipidomic conservation within species and highlights the biological distinctiveness of different milk sources at the lipidomic level—an insight valuable for species classification and authenticity verification.

In ruminants, dietary lipids are extensively hydrolyzed and unsaturated fatty acids are biohydrogenated in the rumen, resulting in high SFA levels in milk [29]. In line with this, buffalo milk showed the most energy-dense lipid profile, enriched in long-chain saturated TG and WE, along with high protein content [30], making it ideal for high-fat dairy applications such as ghee or butter [31]. Goat milk, by contrast, exhibited moderate levels of medium-chain fatty acids (MCFAs), including caproic (C6:0) and caprylic acid (C8:0) [32], which are known for better digestibility and metabolic benefits. Consequently, ruminant milk (e.g., from Holstein cows, Jersey cows, buffalo, yaks, and goats) is characterized by higher SFA content, likely reflecting biomarkers concentrated in SFA. Yak milk, particularly from high-altitude regions, displayed elevated Cer and SM, possibly as adaptations to cold environments for energy storage and thermal protection [33,34]. Of special significance is our finding that LPC (O-16:0) was consistently elevated in Hongyuan yak milk—a registered geographical indication (GI) product in China—compared to ordinary yak milk. Prior traceability studies on Hongyuan yak milk primarily focused on amino acids or small metabolites (e.g., proline, 2-hydroxy-3-methylbutyric acid, L-isoleucine) [35], whereas our study expands this landscape by identifying lipids as promising new GI biomarkers. Milk from camels, classified as pseudo-ruminant due to their three-chambered stomachs, was rich in structural lipids such as PC, PE, SM, and Cer, consistent with previous observations [36,37]. Reportedly, camel milk contains abundant phospholipids, including PC, PE, SM, PI, and PS, comprising up to 1% of total lipids [36]. These lipids are key constituents of the milk fat globule membrane (MFGM), which modulates milk’s nutritional and biological performance [38]. The small fat globule size of camel milk (1.1–2.1 µm) compared to other milks enhances its surface area-to-volume ratio, potentially improving lipid digestibility and bioavailability. These compositional and structural traits support camel milk’s suitability for functional foods targeting immune and cognitive health [29].

### 4.3. Non-Ruminant Milks: PUFA-Rich but Lipid-Light

In sharp contrast to ruminant milk, mare and donkey milk—both non-ruminants—were lipid-light but PUFA-rich. Both milks had markedly higher levels of PUFAs, especially linoleic acid (C18:2) and α-linolenic acid (C18:3), and lower levels of SFAs and MUFAs [39]. These fatty acid patterns closely resemble those in human milk, and the SFA:MUFA:PUFA ratio (~1:1:1) aligns well with FAO/WHO dietary recommendations [40]. In addition, long-chain PUFAs such as docosahexaenoic acid (DHA) and eicosapentaenoic acid (EPA) were detected [28,41], which are known to support cardiovascular and neural health [42].

Moreover, both mare and donkey milk contain low levels of allergenic proteins and exhibit lipid profiles similar to those of human milk [43], making them promising alternatives for infants with a cow milk protein allergy (CMPA) [40,44]. Among them, mare milk is particularly notable for its lower allergenic potential and a lactose content more closely aligned with that of human milk [45]. Additionally, the distribution of TG and DG in mare milk closely resembles that of human milk, characterized by lower concentrations of stearic and palmitic acids and a higher proportion of PUFA. From a processing standpoint, a high SFA content can enhance product stability and prolong shelf life by reducing oxidative rancidity. However, excessive SFA intake has been associated with an increased risk of cardiovascular diseases. In contrast, although high PUFA levels confer various health benefits, they also make the milk more prone to oxidative degradation during storage and processing [46], a concern that has been echoed in industry feedback. Therefore, maintaining product stability remains a key challenge in mare milk processing, necessitating strategies to minimize lipid oxidation. Currently, the high levels of C18:2 (linoleic acid) and C18:3 (α-linolenic acid) in mare milk, along with its unique esterification patterns at the Sn-2 and Sn-1,3 positions, have contributed to its use as a functional ingredient in traditional Mongolian cosmetic products [43]. While lipidomics studies on equine milk remain limited, our findings offer foundational insights for valorizing these underutilized dairy resources in modern food and health sectors.

### 4.4. Methodological and Scientific Advancements

Compared with previous LC-MS-based milk lipidomics studies (Table 1), our work provides several novel contributions: a. Expanded species coverage: The inclusion of underrepresented milks such as donkey, mare, and regional variants (camel1/2, yak1/2) enables a more holistic comparison of milk lipids. b. Geographical differentiation: This is the first report using lipidomics to distinguish regional variants within the same species (e.g., Hongyuan vs. regular yak milk), highlighting the regulatory potential of lipid biomarkers for GI protection. c. Lipid subclass novelty: The detection of rare subclasses (e.g., ACCa, PFAA, WE) in species such as buffalo and donkey milk enriched the current milk lipidomic landscape. d. Updated tools: The use of UHPLC-HRMS and LipidSearch 5.0 improved coverage and classification accuracy, offering advantages over earlier platforms.

These advances support not only the nutritional characterization of milk but also have implications for food safety, labeling, and quality assurance.

### 4.5. Implications for Dairy Product Development and Challenge

Our results provide practical insight for tailoring dairy products based on lipidomic profiles: High-TG, SFA-rich milks (e.g., buffalo, yak) suit applications such as cream, butter, or cheese. Low-lipid, PUFA-rich milks (e.g., donkey, mare) are ideal for hypoallergenic formulas and clinical diets. Camel milk, with its functional phospholipids and sphingolipids, may serve in cognitive or immune-enhancing products. Region-specific markers (e.g., LPC (O-16:0) in Hongyuan yak milk) open opportunities for premium GI branding and anti-fraud strategies. These insights can guide innovation in dairy processing and add value to underutilized milk resources. Despite its comprehensiveness, this study has several limitations. First, the functional roles of specific lipids (e.g., LPC, SM, Cer) were inferred based on known biochemical pathways and the literature, but require experimental validation. Second, lipidomics alone may not capture the full nutritional or sensory profile of milk; future work should integrate proteomics, glycomics, and microbiomics for a holistic view. Third, milk samples were obtained from specific farms and regions, and may not represent global variation. Broader sampling across seasons, breeds, and geographies will enhance the generalizability and regulatory utility of lipid biomarkers.

Additionally, we acknowledge that certain low-abundance or highly hydrophilic lipid subclasses may remain underrepresented due to the limitations of a single extraction and LC-MS method. While our optimized workflow improved the detection of major functional lipids (TG, DG, PL, SM, Cer), future studies employing orthogonal chromatographic techniques (e.g., HILIC or supercritical fluid chromatography) [47,48], alternative ionization strategies (e.g., ion mobility or MALDI) [49], and targeted MS/MS approaches could further enrich lipidome coverage and structural resolution, particularly for polar or signaling lipids that may have regulatory or health-related functions.

## 5. Conclusions

This study presents a comprehensive untargeted lipidomic profiling of ten milk types derived from eight animal species using UHPLC-HRMS combined with LipidSearch 5.0, identifying 640 lipid species spanning major lipid classes. The results revealed clear interspecies and moderate intraspecies lipidomic variation, with ruminant milks such as buffalo and yak characterized by high levels of TG and SFA, making them suitable for high-energy or traditional dairy products. Goat milk exhibited a balanced lipid composition rich in MCFA and Cer, while Hongyuan yak milk—registered as a GI product—was distinguished by elevated levels of LPC (O-16:0), highlighting the potential of lipidomics in origin authentication. Non-ruminant milks, particularly mare and donkey milk, demonstrated low total lipid content but were enriched in health-promoting PUFA, notably C18:2 and C18:3, along with favorable esterification patterns and low allergenicity, supporting their potential use in infant formulas, hypoallergenic diets, or functional beverages. Camel milk stood out for its high phospholipid and sphingolipid content, along with smaller fat globule size, indicating improved digestibility and suitability for cognitive and immune-supporting formulations. Beyond nutritional insights, this study contributes novel lipid markers (e.g., LPC (O-16:0)) and rare lipid subclasses (e.g., WE, ACCa, PFAA), and introduces a lipid-based strategy for distinguishing regional milk variants, offering implications for GI product protection and anti-fraud monitoring.

## Figures and Tables

**Table 1:** Comparison of LC-MS-based lipidomics studies on different milk types.

Reference	Milk Type(s)	Platform and Column	Lipid Identification	Lipid Species	Highlighted Features
Li et al., 2017 [4]	Goat, soy, and cow	UPLC-Q-Exactive Orbitrap, C18	Lipidsearch 4.0	370	Clear differences between plant-based and animal milk lipids.
Li et al., 2020 [20]	Bovine colostrum, mature milk	UHPLC-QTOF-MS, C18	Lipid Analyzer	335	Covered different lactation stages; practical implications for nutritional quality evaluation.
Wang et al., 2023 [19]	Human, ewe colostrum	UHPLC-QTRAP-MS, C30	Lipid Maps	1004	Detected a high number of lipid species using a wide-coverage platform.
Imperiale et al. [12]	Cow	UPLC-Q-Exactive Orbitrap, C18	Lipid Maps	Optimized TG profiling	Developed TG isomer identification strategy.
Zhao et al., 2022 [27]	Human, cow, goat, sheep, camel	UHPLC-Q-Exactive Orbitrap, C18	Lipidsearch 4.1	826~918	Revealed significant differences between human and ruminant milk lipids; relevance to infant formula development.
Wu et al., 2023 [29]	Mare, donkey, camel, yak, pig, human	UHPLC-Q-Exactive Plus, C18	Lipidsearch 4.2	2585	Broad cross-species comparison with limited subgroup analysis.
Present Study	Camel, mare, donkey, goat, buffalo, yak, Jersey, Holstein	UHPLC-Q-Exactive Plus, C18	Lipidsearch 5.0	640	Featured the broadest species coverage and revealed a wide spectrum of lipid subclasses, including rarely reported ones.

## Data Availability

The original contributions presented in this study are included in the article/Supplementary Materials. Further inquiries can be directed to the corresponding author.

## Supplementary Materials

The following supporting information can be downloaded at: https://www.mdpi.com/article/10.3390/foods14122068/s1, Table S1: Data from the untargeted lipidomics analysis, expressed as peak areas.

## Abbreviations

The following abbreviations are used in this manuscript:

FA	fatty acids
GL	glycerolipids
GP	glycerophospholipids
SP	sphingolipids
ST	sterol lipids
SL	saccharolipids
PR	prenol lipids
PK	polyketides
MCT	medium-chain triglycerides
MFGM	milk fat globule membranes
SFA	saturated fatty acids
HRMS	high-resolution mass spectrometry
HPLC-MS	High-performance liquid chromatography—mass spectrometry
MALDI-TOF MS	Matrix-assisted laser desorption ionization—time of flight mass spectrometry
GC-MS	gas chromatography—mass spectrometry
UHPLC-HRMS	ultra-high-performance liquid chromatography—high-resolution mass spectrometry
QC	quality control
PCA	principal component analysis
LOD	limit of detection
ACCa	Acyl carnitine
Cer	Ceramide
Ch	Cholesterol
DAP	Dialkyl phthalates
DG	Diglyceride
Hex1Cer	Hexosyl ceramide
Hex2Cer	Dihexosyl ceramide
LBPA	Monoacylglycerophosphomonoradylglycerols
LPC	Lyso phosphatidylcholine
LPE	Lyso phosphatidylethanolamine
MG	Monoglyceride
MGDG	Monogalactosyl diacylglycerol
PA	Phosphatidic acid
PC	Phosphatidylcholine
PE	Phosphatidylethanolamine
PFAA	Primary amides
PG	Phosphatidylglycerol
PI	Phosphatidylinositol
PS	Phosphatidylserine
SM	Sphingomyelin
TG	Triacylglycerol
WE	Wax ester
PLS-DA	partial least squares—discriminant analysis
PUFA	long-chain polyunsaturated fatty acids
DHA	docosahexaenoic acid
EPA	eicosapentaenoic acid
MUFA	monounsaturated fatty acids
CMPA	cow milk protein allergy
MCFA	medium-chain fatty acids
